# Which Shiftwork Pattern Is the Strongest Predictor for Poor Sleep Quality in Nurses?

**DOI:** 10.3390/ijerph192113986

**Published:** 2022-10-27

**Authors:** Kampanat Wangsan, Naesinee Chaiear, Kittisak Sawanyawisuth, Piyanee Klainin-Yobas, Kanjana Simajareuk, Watchara Boonsawat

**Affiliations:** 1 Department of Community, Family and Occupational Medicine, Faculty of Medicine, Khon Kaen University, Khon Kaen 40002, Thailand; 2 Department of Community Medicine, Faculty of Medicine, Chiang Mai University, Chiang Mai 50200, Thailand; 3 Department of Medicine, Faculty of Medicine, Khon Kaen University, Khon Kaen 40002, Thailand; 4 Alice Lee Center for Nursing Studies, Yong Loo Lin School of Medicine Clinical Research Centre, National University of Singapore, Singapore 119077, Singapore; 5 Nursing Division, Srinagarind Hospital, Faculty of Medicine, Khon Kaen 40002, Thailand

**Keywords:** backward, sleep, risk factors, nurse, shiftwork

## Abstract

Shiftwork is related to an increased risk of several diseases, including gastric ulcers, myocardial infarction, and diabetes. Several shiftwork patterns are related to poor sleep quality, such as a quick returns or extended shifts. This study aimed to find the shiftwork patterns strongly associated with poor sleep quality amongst nurses. A cross-sectional analytical study was conducted among nurses working for at least one month. The sub-groups were the good sleep quality group (n = 150) and the poor sleep quality group (n = 472). Eligible participants were asked to complete a self-reported questionnaire comprising personal characteristics, job characteristics, shiftwork characteristics, and sleep quality. Factors associated with poor sleep quality were determined using logistic regression analysis. Two factors associated with poor sleep quality remained in the final model: viz., depression and backward rotational shift. The only independent factor for poor sleep quality was a backward rotational shift with an adjusted odds ratio (95% CI) of 1.946 (1.344, 2.871). In conclusion, compared with other shift patterns, backward shiftwork was the most significant factor associated with poor sleep quality and should be avoided.

## 1. Introduction

Although shiftwork helps manage 24 h work operations, it has measurably disruptive effects on the quality of family life, social life, and work efficiency [1]. Regarding health effects, shift workers demonstrated a greater risk of myocardial infarction than non-shiftwork workers [2]. Shiftwork is also associated with body weight increase, perhaps because of increased food intake and poor-quality fast food [3].

The International Agency for Research on Cancer (IARC) has classified shiftwork as a 2A carcinogen. Lahti et al. reported that shiftwork increased the risk of non-Hodgkin’s lymphoma [4]. Shiftwork or extended-hours workers also had an increased risk of work accidents [5], and those who either worked less than 5 years or more than 15 had a significantly greater risk of workplace accidents than non-shiftwork workers [6].

Shiftwork is necessary for some occupations, including nurses; notwithstanding, it is related to an increased risk of several diseases (i.e., gastric ulcers, myocardial infarction, and diabetes) [7,8,9]. Shift workers were at significant risk of myocardial infarction and stroke (relative risk 1.23 and 1.05 times greater, respectively) [8]. A study from Turkey reported that shift nurses had a lower quality of life according to the SF-36 MCS (35.5 vs. 42.5) and lower life satisfaction (16.4 vs. 19.9) compared with daytime nurses [10].

Several studies revealed the factors associated with the quality of sleep [11], such as insomnia, pain, drug use, alcoholic beverages, caffeinated drinks [12], obstructive sleep apnea, depression, [13] and stress [14]. Relatedly, shiftwork was a critical negative factor related to poor quality of sleep [15,16,17,18]. 

Shiftwork is related to poor sleep quality, with an odds ratio of 3.975 [17]. A report from Taiwan reported that 96% of female nurses complained of poor sleep quality [19], which may relate to alertness and errors during working hours [20,21]. Nurses who had long working hours committed more medication errors (4% vs. 3%; *p* < 0.001) than those who worked fewer hours [21]. Our previous study found several shiftwork patterns among nurses working at a university hospital, including day shift plus overtime (6.1%), day and evening shift (3.7%), day and night shift (0.7%), and rotational shift (89.5%) [22]. The majority of participants had quick returns shifts (88.0%). The median quick returns shift was 11 times monthly (IQR 7–13). They were all irregular directional shifts with fast rotations. The researchers divided the nurses into 3 groups (viz., primarily forward, primarily backward, and evenly split between backward and forward). Most nurses worked in a primarily backward rotation pattern (479; 90.2%), followed by a primarily forward direction (6.4%). There are several shiftwork patterns related to poor sleep quality [15,16]. Having a quick returns shiftwork pattern of over 30 times per year is 2.53 times more likely to be associated with poor sleep quality, while an extended shift of 12.5 h or more has 2.4 times greater risk of poor sleep quality. 

Nursing is an occupation of great responsibility for the well-being of patients while targeting on the importance of health maintenance and the treatment of illness. Hospital nurses work in a shift system, putting them at risk of poor sleep quality. Insufficient sleep may be related to alertness and errors while at work [15,16,20,21]. Several previous studies documented the unavoidable health effects associated with shiftwork in healthcare processes. Good scheduling, without improper shiftwork patterns, would help reduce adverse effects; however, there is limited data on which shiftwork pattern has the most substantial impact on poor sleep quality. Therefore, the current study aimed to explore the relationship between the different shiftwork patterns and sleep quality. 

## 2. Materials and Methods

### 2.1. Design

This cross-sectional analytical study was conducted at Khon Kaen University Hospital, Khon Kaen, Thailand. 

### 2.2. Study Population

The target population were nurses who had worked for at least one month and were willing to participate in the study. Those on maternal leave, sabbatical leave, or who provided incomplete clinical data were excluded. There were two study groups: the good sleep quality group (n = 150) and the poor sleep quality group (n = 472). The study period was between March and May 2018. This study was a part of a previously published article [22].

### 2.3. Tools

The researchers constructed a self-reported questionnaire comprising personal characteristics, job characteristics, characteristics of shiftwork, and sleep quality. The nurses in our hospital had three working patterns: fixed shift, rotational shift, and others. The fixed pattern comprised four patterns: regular working hours (or no shifts), day and afternoon shifts, day and night shifts, and day shifts with overtime. Rotational shiftwork is defined by direction: no rotation, forward, backward, or equal. Forward rotational shift is a clockwise rotation of more than 50% of shifts, while backward rotational shift is a counterclockwise rotation of more than 50% of shifts. Equal shifts had as many forward and backward shifts. There were two additional working patterns, including a quick returns and extended shifts. Quick returns is defined by the duration between shifts of less than 11 h, while an extended shift is defined by working more than an 8 h shift. These two patterns were counted and reported per month. The sleep quality, an outcome, was measuredp using the Pittsburgh Sleep Quality Index (PSQI) [23]. The total score of PSQI of five or over indicated poor sleep quality, while a total score of 0–5 indicated good sleep quality.

### 2.4. Statistical Analysis

Eligible participants were categorized into two groups: either good or poor sleep quality. The studied variables were compared between these two groups using descriptive statistics. The factors associated with poor sleep quality were determined through a logistic regression analysis. 

A univariate logistic regression analysis was used to calculate the unadjusted odds ratio with a significant *p* value. Those factors with a significant *p* value in the univariate logistic regression analysis were put into a multivariate logistic regression analysis: stepwise method. Multivariate logistic regression analyses were also computed, by adding shiftwork patterns to the final model. The final models predictive of poor sleep quality were tested for goodness of fit using the Hosmer–Lemeshow method. The results were reported as means (SD), numbers (percentages), unadjusted odds ratios (95% confidence interval), and adjusted odds ratios (95% confidence interval). All analyses were performed using STATA software, version 10.1 (College Station, TX, USA).

## 3. Results

The respective baseline and job characteristics of those with good and poor sleep quality are shown in Table 1. In general, there were no statistical differences in personal and work characteristics except in age and work experience. The poor sleep quality group was significantly younger (34.3 vs. 37.3 years; *p* 0.004) and had less work experience (10.5 vs. 12.4 years; *p* 0.033) than those in the good sleep quality group (Table 1).

Four different factors were significantly associated with the shiftwork patterns, distributed between those with good and poor sleep quality (Table 2). The poor sleep quality group had a higher average number of quick returns patterns than the good sleep quality group (7.9 vs. 6.4 times/month; *p* 0.002). The good sleep quality group had a higher proportion of non-shift patterns (26.7% vs. 15.0%; *p* 0.002) and no night shift pattern (36.7% vs. 23.7%; *p* 0.003) compared to the poor sleep quality group. Finally, the poor sleep quality group had a higher proportion of rotational shifts than the good sleep quality group (75.9% vs. 62.7%; *p* 0.002); mainly, the backward direction (71.7% vs. 55.7%; *p* 0.001).

Two factors associated with poor sleep quality remained in the final model: depression and backward rotational shift (Table 3). Only backward rotational shift was an independent factor for poor sleep quality with an adjusted odds ratio (95% confidence interval) of 1.946 (1.344, 2.871). The Hosmer–Lemeshow Chi-square of the final model was 1.77 (*p* 0.413). After adding shiftwork patterns to the final model, backward rotational shift remained significant with an adjusted odds ratio of 8.189 (Table 4). The Hosmer–Lemeshow chi-square of this model was 4.63 (*p* 0.795).

## 4. Discussion

The current study found that nurses at a university hospital had a high prevalence of poor sleep quality (75.9%). The rate was somewhat higher than reported in a previous report from China (62.1%) [24] The current study may have a higher poor sleep quality rate because there were several shiftwork patterns, particularly rotational shiftwork (452 persons: 72.7%), as previously reported [22]. Rotational shiftwork is reported to affect the circadian rhythm, with an odds ratio of 1.456–2.348 times for poor sleep quality [23,25,26,27,28,29]. Rotational shiftwork is usually divided according to two criteria: viz., speed of rotation and direction of rotation. In a previous study, the speed of rotation over nine days predicted the least-mean drowsiness scale, which was based on how long the subject took to adjust to new circadian rhythms. As well as changing rotation every three days, drowsiness occurred when trying to work before the circadian rhythm could adjust to the new working time. By contrast, a one-day quick returns had no effect on circadian rhythms, nor did changing rotation every 6 days, as the circadian rhythm simply started adjusting [19]. Meanwhile, the study from China enrolled nurses working night shift consecutively, leading to a more familiar sleep pattern. However, the poor sleep quality rates among nurses were very high compared to other occupations in the USA (30.7%) [30]. These findings suggest different rates of poor sleep quality among occupations.

Several studies have shown that shiftwork patterns have a significant effect on sleep quality, including quick returns, extended shifts, and rotational shifts [16,25,31,32,33]. In addition, extending the shiftwork effect could influence executive function [34].

This study found that backward rotational shiftwork resulted in the strongest association with poor sleep quality compared to the other shift patterns (Table 3 and Table 4). Shon et al. found that backward rotation had a significant association with poor sleep quality compared with forward rotation regardless of sex [23]. The adjusted odds ratio of backward rotation was 1.95, which was comparable to the current study (1.946). Taken together, these findings may explain how adaptation of the circadian rhythm for shift pattern, as previously reported in jet lag situation, may impact upon sleep. Forward rotation requires a more rapidly adapted phase delay, while backward rotation requires slower adaptation as a phase advance [35,36].

This study had some limitations. First, this was a single-site study at a university hospital, but it had a large sample size and specificity. Second, some factors were not evaluated (i.e., sleep apnea). Finally, data received from the participants were self-reported, resulting in information bias. 

## 5. Conclusions

In conclusion, poor sleep quality was prevalent among nurses working at a university hospital. Backward shiftwork was the strongest factor associated with poor sleep quality compared with other shift patterns. The backward pattern should thus be minimized in order to improve sleep quality for nurses. The limitation of this study was the cross-sectional design, which resulted in causal relationships not being able to be confirmed. To help with shift management, an interventional study using different patterns (i.e., different patterns and measuring the quality sleep) is recommended.

## Figures and Tables

**Table 1:** Baseline characters and job characters of nurses categorized by sleep quality.

Factor	Good Sleep Quality (n = 150) n (%)	Poor Sleep Quality (n = 472) n (%)	*p*-Value
Age, years *	37.30 (10.81)	34.38 (9.98)	0.004
Male sex	6.00 (4.00)	19.00 (4.03)	0.603
Married	69.00 (46.00)	192.00 (40.68)	0.256
Children	58.00 (38.67)	160.00 (33.90)	0.326
Number of children *	0.62 (0.81)	0.52 (0.80)	0.27
Working experience, year	12.36 (9.97)	10.46 (9.28)	0.033
Obesity	29.00 (19.33)	76.00 (16.10)	0.381
Smoking	0	2.00 (0.42)	0.999
Alcohol	1.00 (0.67)	3.00 (0.64)	0.999
Caffeine	55.00 (36.67)	175.00 (37.08)	0.999
Studying postgraduate level	5.00 (3.33)	18.00 (3.81)	0.999
Depression	27.00 (18.00)	122.00 (25.85)	0.061
High risk for OSA **	16.00 (10.67)	66.00 (13.98)	0.334
Chronic illness	34 (22.67)	118.00 (25.00)	0.587
Job nurse, not boss	137 (91.33)	451.00 (95.55)	0.062
Job type			0.972
Healthcare	126.00 (84.00)	395.00 (83.69)	
Management	3.00 (2.00)	11.00 (2.33)	
Mixed	21.00 (14.00)	66.00 (13.98)	
Location: IPD	119.00 (79.33)	401.00 (84.96)	0.128
Salary: not enough	15.00 (10.00)	50.00 (10.59)	0.999
Extra jobs	18.00 (12.00)	61.00 (12.92)	0.888
In hospital	13.00 (8.67)	40.00 (8.47)	0.999
Outside hospital	5.00 (3.33)	21.00 (4.45)	0.646
Extra work activity	3.00 (2.00)	8.00 (1.68)	0.732

Note. Data presented as number (percentage) except * indicated mean (SD); ** OSA: obstructive sleep apnea by Berlin questionnaire; IPD: inpatient department.

**Table 2:** Characters of shift patterns of nurses categorized by sleep quality.

Factor	Good Sleep Quality (n = 150) n (%)	Poor Sleep Quality (n = 472) n (%)	*p*-Value
Fixed shift	56 (37.33)	114 (24.15)	
Non-shift	40 (26.67)	71 (15.04)	0.002
Day and afternoon shift	6 (4.00)	15 (3.18)	0.608
Day and night shift	1 (0.67)	2 (0.42)	0.564
Day and over time shift	9 (6.00)	26 (5.51)	0.839
Rotational shift, n	94 (62.67)	358 (75.85)	0.002
Forward	8 (5.37)	22 (4.71)	0.827
Backward	83 (55.70)	335 (71.73)	0.001
Equal	3 (2.01)	2 (0.43)	0.094
Others			
No. of quick returns/month *	6.39 (6.00)	7.95 (5.99)	0.002
No. of extend shift/month *	0.74 (2.42)	0.61 (2.08)	0.125

Note: Data presented as number (percentage) except * indicated mean (SD).

**Table 3:** Factors associated with poor sleep quality in nurses by stepwise method.

Factor	Unadjusted Odds Ratio (95% Confidence Interval)	Adjusted Odds Ratio (95% Confidence Interval)
Depression	1.587 (0.997, 2.527)	1.572 (0.984, 2.513)
Backward	1.973 (1.352, 2.881)	1.946 (1.344, 2.871)

**Table 4:** Factors associated with poor sleep quality in nurses by adding various shift patterns.

Factors	Unadjusted Odds Ratio (95% Confidence Interval)	Adjusted Odds Ratio (95% Confidence Interval)
Age	0.973 (0.956, 0.990)	0.972 (0.934, 1.011)
Work experience	0.979 (0.961, 0.998)	1.018 (0.982, 1.056)
Non-shift	0.486 (0.313, 0.756)	0.656 (0.283, 1.517)
Rotational	1.870 (1.263, 2.769)	0.146 (0.020, 1.055)
Forward	0.867 (0.378, 1.991)	5.302 (0.748, 37.549)
Backward	1.973 (1.352, 2.881)	8.189 (1.317, 50.901)
Quick returns	1.045 (1.012, 1.078)	0.995 (0.944, 1.049)
Extended shift	0.975 (0.900, 1.055)	0.980 (0.889, 1.079)
Depression	1.587 (0.997, 2.527)	1.602 (0.994, 2.584)
